# A Multi-Scale U-Shaped Convolution Auto-Encoder Based on Pyramid Pooling Module for Object Recognition in Synthetic Aperture Radar Images

**DOI:** 10.3390/s20051533

**Published:** 2020-03-10

**Authors:** Sirui Tian, Yiyu Lin, Wenyun Gao, Hong Zhang, Chao Wang

**Affiliations:** 1 Department of Electronic Engineering, School of Electronic and Optical Engineering, Nanjing University of Science and Technology, Nanjing 210094, China; 2 Department of Electrical and Computer Engineering, University of California, Riverside, Riversidem, CA 92521, USA; ylin170@ucr.edu; 3 College of Computer and Information, Hohai University, Nanjing 211100, China; gao_wy@les.cn; 4 Key Laboratory of Digital Earth Science, Institute of Remote Sensing and Digital Earth, Chinese Academy of Sciences, Beijing 100094, China; zhanghong@radi.ac.cn (H.Z.); wangchao@radi.ac.cn (C.W.); 5 College of Resources and Environment, University of Chinese Academy of Sciences, Beijing 100049, China

**Keywords:** multi-scale representation learning (MSRL), pyramid pooling module (PPM), compact depth-wise separable convolution (CSeConv), convolution auto-encoder (CAE), object classification, synthetic aperture radar (SAR)

## Abstract

Although unsupervised representation learning (RL) can tackle the performance deterioration caused by limited labeled data in synthetic aperture radar (SAR) object classification, the neglected discriminative detailed information and the ignored distinctive characteristics of SAR images can lead to performance degradation. In this paper, an unsupervised multi-scale convolution auto-encoder (MSCAE) was proposed which can simultaneously obtain the global features and local characteristics of targets with its U-shaped architecture and pyramid pooling modules (PPMs). The compact depth-wise separable convolution and the deconvolution counterpart were devised to decrease the trainable parameters. The PPM and the multi-scale feature learning scheme were designed to learn multi-scale features. Prior knowledge of SAR speckle was also embedded in the model. The reconstruction loss of the MSCAE was measured by the structural similarity index metric (SSIM) of the reconstructed data and the images filtered by the improved Lee sigma filter. A speckle suppression restriction was also added in the objective function to guarantee that the speckle suppression procedure would take place in the feature learning stage. Experimental results with the MSTAR dataset under the standard operating condition and several extended operating conditions demonstrated the effectiveness of the proposed model in SAR object classification tasks.

## 1. Introduction

As the vital task of object classification with synthetic aperture radar (SAR) images, feature engineering intends to obtain robust representations of intrinsic properties to distinguish various targets in high-resolution radar images. Although numerous hand-designed features have been proposed to represent both the spatial and electromagnetic characteristics of targets over the past decades, feature learning is still a challenging task for SAR-based automatic target recognition (SAR ATR) applications.

In general, the traditional hand-designed features include two categories: the generalized features [1,2,3] and the SAR-specialized features [4,5,6,7]. The former ones involve features from other domains considering little of the characteristics of SAR imagery, while the latter ones refer to those designed for specific SAR ATR tasks. Despite their high accuracy while dealing with the benchmark or specific SAR dataset, all these handcrafted features have certain obstacles. A major drawback is the requirement of detailed prior knowledge about the potential applications that are sometimes unavailable. Another obstacle is that many features, especially those based on scattering models, hold a series of assumptions for operation conditions (OCs), leading to performance degradation when the assumptions are inconsistent with the OCs. Accordingly, it is necessary to devise new feature learning algorithms which can adaptively learn representations from various data, considering complicated situations.

With the theoretical progress of machine learning, the deep learning (DL) model, which has turned out to be adept at automatically discovering intricate information in high-dimensional raw data [8], has been employed to tackle SAR ATR tasks and achieved the superior performance than hand-designed features. Although the supervised DL models have obtained state-of-the-art results, their requirement of a great many labelled data is the major obstacle in SAR ATR. The labelled benchmarks are too small to train a supervised deep network effectively, and overfitting caused by limited labelled samples is often one of the main causes of performance degradation of the supervised model. To handle this problem, various unsupervised DL models are employed and developed, including the autoencoder (AE) [9,10], the generative adversarial network (GAN) [11,12], and the restricted Boltzmann machine (RBM) [13]. Due to the fact of its simple implementation and attractive computational cost, the AE has widely been used in SAR ATR which minimizes the distortion between the inputs and the reconstructions to guarantee that the mapping process preserves the information of the inputs.

In earlier works, the autoencoder was utilized to derive refined representations from the predefined features or preprocessed images before feeding them into a traditional classifier such as the softmax or the support vector machine (SVM) [14,15,16,17,18,19,20]. In Reference [14], Geng et al. presented a deep convolution AE (CAE) for SAR image classification. Two kinds of handcrafted features, a gray-level co-occurrence matrix (GLCM) and the Gabor filter banks, were jointly fed into the CAE. The learned representation was subsequently fed to a softmax classifier for land-cover classification. In Reference [15], the geometric parameters and the local texture features were combined to train a stacked AE (SAE) for vehicle classification in SAR images. Gleich and Planinšic [16] estimated the log commulants of SAR data patches via the dual-tree oriented wavelet transform and input them into an SAE to derive representations for scene patch categorization. Zhang et al. [17] devised a framework to learn the robust representation of polarimetric SAR (PolSAR) data based on the spatial information, which was characterized by the spatial distance to the central pixel. Their framework was subsequently improved in [18] with a multi-scale strategy in which the spatial information was obtained by taking neighborhood windows of different scales before the stacked sparse AE (SSAE) was applied to extract features at different scales for land cover classification in PolSAR images. Hou et al. [19] devised a PolSAR image classification method based on both the multilayer AE (MLAE) and the superpixel trick. The superpixels produced by the Pauli decomposition to integrate contextual information of the neighborhood was refined by an MLAE to generate a robust representation. Chen and Jiao [20] fed the discriminative feature extracted by the multilayer projective dictionary into an SAE to realize the nonlinear relationship between the elements of feature vectors in an adaptive way.

To further promote the performance in representation learning, various models based on the AE framework have also been utilized [21,22,23,24]. In Reference [21], the stacked contractive AE (SCAE) was utilized to extract temporal characteristics from superpixels for change detection in SAR images which was restricted by a contractive penalty with the Frobenius norm of the Jacobian. Xu et al. [22] developed an improved variational AE (VAE) based on the residual network to draw latent representations for vehicle classification in SAR images. Song et al. [23] devised an adversarial autoencoder neural network (AAN) to learn intrinsic characteristics and generate new samples at different azimuth angles by adversarial training. Kim and Hirose [24] proposed a quaternion autoencoder and a quaternion self-organizing map (SOM) for PolSAR image classification. The quaternion AE was introduced to extract representations based on the natural distribution of PolSAR features. The extracted features were classified by the quaternion SOM in an unsupervised manner, by which new and more detailed land categories could be discovered. In Reference [25], a deep bimodal AE was proposed for land cover classification by fusing the SAR and the multispectral images. The bimodal AE provided independent encoding modalities in the front part to learn the features of SAR data and fused the feature of each modality with shared representation layers to obtain the representations for classification.

Despite the explosive growth of unlabeled SAR images with the development of high-resolution SAR systems, the training dataset (even the unlabeled benchmarks) available for specific tasks or targets are limited and incomplete. To handle this problem, model transferring is recommended as another solution to improve the representation learning capability with small sample size and limited training resources. Huang et al. [26] devised an assembled CNN model that combines a CAE with a CNN, sharing the encoder part of the CAE. The CAE was pre-trained with a large number of unlabeled SAR images, and its encoder part that connected with a fully connected layer was fine-tuned with the limited target patches. Mohammad et al. [27] proposed a domain adaptation algorithm to transfer knowledge from the earth observation (EO) domain to the SAR domain. They trained two deep encoders coupled through their last layer to map data points from the EO and the SAR domains to the shared embedding space, such that the distance between the distributions of the two domains was minimized in the latent embedding space. In Reference [28], a DL-based workflow was proposed to map forest above-ground biomass by integrating Landsat 8 and Sentinel-1A images with airborne light detection and ranging (LiDAR) data. They demonstrated the advantage of a stacked sparse autoencoder network in comparison to other prediction techniques. De et al. [29] proposed an AE-based technique for urban area classification in PolSAR images which leveraged a synthetic target database for data augmentation (DA). The synthetic dataset obtained by rotation and collation was fed to an SAE to generate a compact representation of the information in the augmented dataset. Although these model transferring methods have alleviated overfitting of DL models caused by small datasets and achieved the state-of-the-art performance, it is quite difficult to design the transferring schemes for specific SAR ATR tasks in various extended operation conditions (EOCs). Besides, the selection of the pre-trained model and the natural image dataset for information transferring will also greatly affect the performance of these approaches. If there is a great difference between the natural images and the objective SAR dataset, the representation learning capability of the transferred models will suffer serious degradation.

Another way to promote the performance of the AE models with a limited training dataset is to incorporate prior knowledge in the model with certain regularization terms and task-specific cost functions. The training process of the AE refers to estimating the trainable parameters of the model and can be achieved by optimizing the objective function consisting of a reconstruction loss and certain regularization terms [9]. In References [30,31], the supervised information was embedded in the cost function by designing label-related regularization terms. Deng et al. [30] devised a Euclidean distance restriction in the cost function which encouraged the intra-class distance of features to be a small value near zero and the inter-class distance to be close to a constant. A similar idea was applied in Reference [31], where the objective function was tuned according to the SAR ATR task. The authors devised a regularization term based on the modified triplet loss that combines the semi-hard triplet loss with the intra-class distance penalty to learn discriminative features with a small intra-class divergence and a large inter-class divergence. In References [32,33,34], the task-specialized prior knowledge was embedded in the objective function of the AE-based model. Xie et al. [32] proposed a new type of AE and CAE with a modified objective function according to the task of PolSAR image classification, where the distortion of the reconstructed data to the inputs was measured by the Wishart distance instead of the ordinary mean square error (MSE) or cross-entropy. Similarly, Wang et al. [33] devised a hybrid AE for land cover classification, where the Wishart distance and Euclidean distance were jointly applied to evaluate the reconstruction error between the input and the output according to the distribution of PolSAR data matrix. In Reference [34], Li et al. proposed a stacked fisher AE for change detection, where the ratio difference image (RDI) of multi-temporal SAR images was used as the input and the distribution of the RDI was introduced to construct the objective function with sparsity regularization.

Although these AE-based models have developed an effective way to learn the robust representation via an unlabeled SAR dataset and achieved competitive results, the performance of most of these models is still slightly inferior to their supervised counterparts [35,36,37,38,39] and some handcrafted features [4,5,7] that are based on the electromagnetic scattering models. The major reasons include the following:

(1) Most of these models learn representations at a large single scale with the hierarchical structure. However, without using local and detailed discriminative information at multiple scales, the classification performance of the features learned by these methods is limited;
(2) Most of the models are optimized according to the minimum reconstruction deviation criterion, importing useless information of speckle in the learned feature and diluting the discriminative features that could benefit classification and ATR tasks;
(3) Small incomplete training benchmarks in SAR ATR limit the application of complicated and deeper DL model due to the large number of trainable parameters and overfitting arising therefrom.

In this paper, a novel unsupervised multi-scale CAE (MSCAE) is proposed which can extract features at different scales and discard useless information of speckle and background clutter. The proposed model provides a framework to learn multi-scale features at two levels: the modality level feature learning achieved by the U-shaped structure and the branch level feature extracted by the pyramid pooling module (PPM). A modified objective function was devised to tackle the performance degradation caused by speckle. The reconstruction loss of the MSCAE was measured between the output and the input filtered by the improved Lee sigma filter (ILSF) [40] to alleviate the influence of serious speckle in SAR images. The structural similarity index metric (SSIM) was employed as the measurement of the reconstruction deviation, taking full advantage of the targets’ characteristics such as the structure and the variation of backscattering intensity. An additional filter regularization term was also incorporated in the objective function that measures the dissimilarity of the encoded features of the raw data and the ILSF filtered inputs, guaranteeing that the speckle suppression procedure occurred during the encoding stage. Moreover, to handle the performance degradation caused by the limited training dataset, a new convolution layer, named compact depth-wise separable convolution (CSeConv) layer, and its deconvolution counterpart (CSeDeConv layer) were also developed to reduce the number of the trainable parameters in the model, alleviating overfitting caused by limited training samples.

The rest of this paper is organized as follows: Section 2 illustrates the key technologies used to build our MSCAE model, including the CSeConv and CSeDeConv, the PPM processing, and the specially designed objective function. Furthermore, the technical details of network topology are also given. Section 3 conducts a series of comparative experiments based on the moving and stationary target acquisition and recognition (MSTAR) dataset [41,42]. The experimental results of the proposed network with SOC and various EOCs are presented. Section 4 concludes our work.

## 2. The Multi-Scale Convolution Auto-Encoder

### 2.1. Overall Structure of the MSCAE

**Figure 1:**
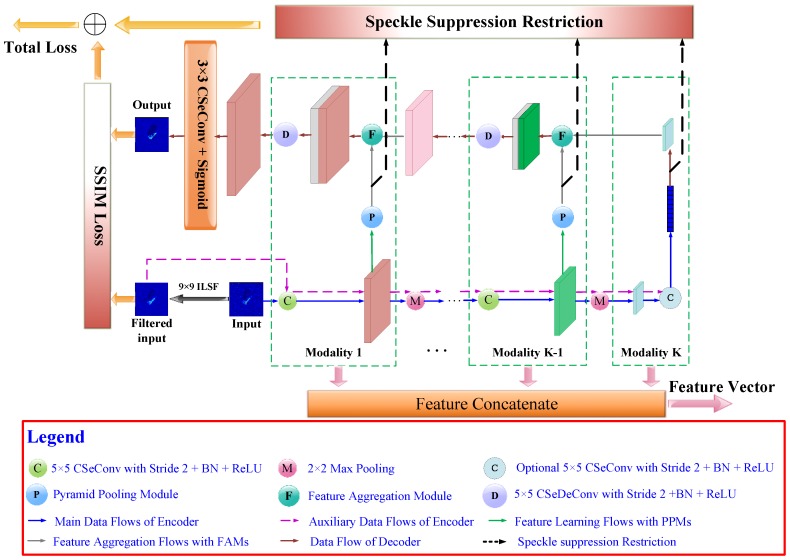
The overall architecture of the U-shaped multi-scale convolution auto-encoder. The feature vector learnt at each modality level will be converted into vector and concatenated to form the feature vector for SAR ATR. CSeConv stands for the compact depth-wise separable convolution layer; BN stands for batch normalization; ReLU stands for the rectified linear unit activation function; CSeDeConv denotes the compact separable deconvolution layer; PPM stands for the pyramid pooling modul; FAM stands for the feature aggregation module; ILSF refers to the improved Lee sigma filter; SSIM refers to the structural similarity index metric.

In this part, we discuss the characteristics and general layout of the proposed MSCAE. As shown in Figure 1, the MSCAE consists of a series of uniform modalities to learn representations and generate the reconstructed feature map at different modality levels. Each modality includes an encoder part and the corresponding decoder part.

In the encoder part of a modality, the CSeConv and the PPM module are applied to learn multi-scale features at branch level. The input feature map will be convolved with a 5×5 CSeConv layer with a stride 2 which means that both the width and the height of the output feature map will be half the size of the input. Subsequently, the batch normalization (BN) and the rectified linear unit (ReLU) activation function will be applied to the convolved feature map. The output feature map will be processed in two branches: one for multi-scale representation learning with the PPM module and the other for the processes in next modality after downsampled by a 2×2 max-pooling layer. It should be noted that at the coarsest modality level, the PPM module is neglected, and the convolved feature map is optional. If the input feature map is larger than 5×5, the convolution layer will be applied. Otherwise, it will also be neglected. The processed feature map will be directly vectorized to form the feature vector of the coarsest level.

In the decoder part of each modality, the feature aggregation module (FAM) is adopted to combine the feature vector learned by the PPM with the feature map reconstructed from the coarser modality level. The combined feature map is convolved by a 5×5 CSeDeConv layer with a stride 2 to upsample the feature map as well as reduce the channel number. At the first modality level, the reconstructed feature map is convolved with a 3×3 CSeConv layer followed by a sigmoid activation function, and the reconstructed image is generated.

The SSIM loss is measured between the reconstructed image and the image filtered by the 9×9 ILSF to diminish the influence of the speckle. Besides, the speckle suppression restriction is also computed to force the speckle suppression taking place at the feature learning procedure. Therefore, an additional auxiliary data flow is fed to the encoder part of the proposed model, where the image filtered by the 9×9 ILSF is encoded with similar modules and parameters at each modality level. The similarity between the feature vectors learned from the unfiltered image and those learned from the filtered one will be compared and summarized to construct the speckle suppression restriction. The weighted sum of the SSIM loss and the speckle suppression restriction forms the objective function of the proposed model. Once the model is trained with the dataset, the encoder part will be utilized to learn representations and the feature vector learned at each modality level will be concatenated to generate the final feature vector for SAR ATR.

### 2.2. Compact Depth-Wise Separable Convolution and the Corresponding Deconvolution

**Figure 2:**
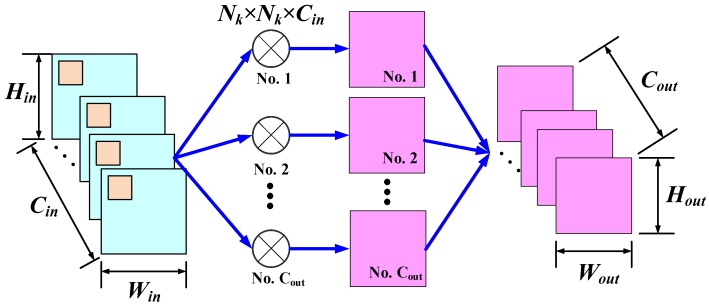
The convolution procedure of a standard convolution layer. Given a $W_{in}\times H_{in}\times C_{in}$ feature map $F_{in}$ with $W_{in}\times H_{in}$ being the spatial size and $C_{in}$ being the number of channels of the input feature map, respectively, the standard convolution layer utilized $C_{out}$ trainable convolution kernels whose size is $N_{k}\times N_{k}\times C_{in}$ to produce the $W_{out}\times H_{out}\times C_{out}$ feature map $F_{out}$ with $W_{out}\times H_{out}$ being the spatial size and $C_{out}$ being the number of channels of $F_{out}$, respectively.

The convolution layer, which is the basic structure in CNNs and CAEs, has the capability of capturing local patterns of input data and generating new representations of jointly encoding space and channel information. As presented in Figure 2, the standard convolution layer [43] creates $C_{out}$ trainable convolution kernels that are convolved with the $W_{in}\times H_{in}\times C_{in}$ input $F_{in}$ to produce a $W_{out}\times H_{out}\times C_{out}$ feature map $F_{out}$. Here, $W_{in}\times H_{in}$ and $W_{out}\times H_{out}$ are the spatial size of the input and the output feature maps, respectively; $C_{in}$ and $C_{out}$ are the channels of $F_{in}$ and $F_{out}$, respectively. The size of each trainable convolution kernel is $N_{k}\times N_{k}\times C_{in}$ with $N_{k}$ being the size of the sliding filter.

In comparison with the fully connected layer, the number of trainable parameters in a convolution layer is much fewer due to the shared convolution kernels, substantially decreasing the computational cost and improving the performance with a small dataset. However, in large-scale deep networks, where the size of the convolution kernels is quite large and the channel number rises rapidly as the depth of the network increases, the high computational cost and overfitting caused by massive trainable parameters are still major causes of performance deterioration. To diminish these problems, various factorized convolution operators are utilized.

**Figure 3:**
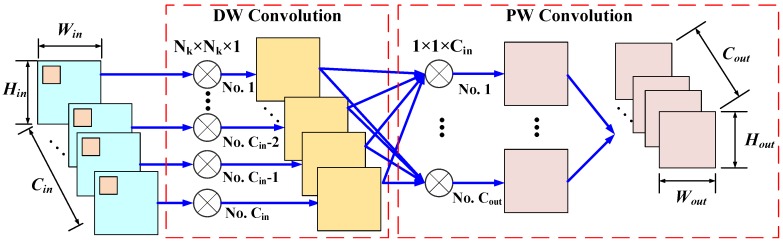
The convolution procedure of the depth-wise (DW) separable convolution (SeConv) layer that decomposes the standard convolution into two steps: the DW convolution and the point-wise (PW) convolution. During the DW convolution process, each channel of the $W_{in}\times H_{in}\times C_{in}$ feature map, $F_{in}$, is convolved with a filter the size of $N_{k}\times N_{k}\times 1$ to generate an intermediate feature map that has $C_{in}$ channels. Subsequently, $C_{out}$ PW filters with the size $1\times 1\times C_{in}$ are adopted to combine the output of the depth-wise layer and produce the final output feature map, $F_{out}$, with the size of $W_{out}\times H_{out}\times C_{out}$

The depth-wise (DW) separable convolution (SeConv) [44], presented in Figure 3, is a typical factorized convolution operator in channel level which factorizes the standard convolution into two steps via the DW convolution and the pointwise (PW) convolution. In the DW convolution step, $C_{in}$ filters with the size of $N_{k}\times N_{k}\times 1$ are applied to every input channel of the $W_{in}\times H_{in}\times C_{in}$ feature map $F_{in}$ and produce the intermediate feature map that has the same number of channels as that of the inputs. Subsequently, the PW convolution utilizes $C_{out}$ filters with the size of $1\times 1\times C_{in}$ to combine the output of the depth-wise layer and produce the final output feature map $F_{out}$.

**Figure 4:**
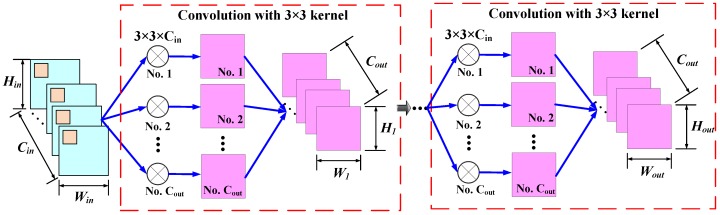
The procedure of the kernel decomposition convolution layer that decomposes the large convolution kernels into M stacked 3×3 filters. Given the $W_{in}\times H_{in}\times C_{in}$ feature map, $F_{in}$, $C_{out}$ small convolution kernels with the size of $3\times 3\times C_{in}$ are applied to generate the first intermediate feature map with the size of $W_{1}\times H_{1}\times C_{out}$. Subsequently, the first intermediate feature map is convolved successively with $C_{out}$ small convolution kernels with the size of $3\times 3\times C_{out}$ for M−1 times, and the output feature map $F_{out}$ with the size of $W_{out}\times H_{out}\times C_{out}$ is produced.

Another factorized convolution layer is the kernel decomposition convolution (DeCConv) layer proposed by Simonyan and Zisserman [45] which decomposes the large convolution kernel into a series of 3×3 small kernels as depicted in Figure 4. Specifically, a large convolution kernel with the size $N_{k}\times N_{k}$ is approximated by M cascaded 3 × 3 filters, where the number of the 3×3 filters is determined by:

(1) $$M=\left(N_{k}-1\right)/2$$

During the convolution procedure with stacked 3×3 filters, activation functions can be employed after each convolution operator. Given the $W_{in}\times H_{in}\times C_{in}$ feature map, $F_{in}$, $C_{out}$ small convolution kernels with the size of $3\times 3\times C_{in}$ are applied to generate the first intermediate feature map with the size of $W_{1}\times H_{1}\times C_{out}$. Subsequently, the first intermediate feature map is convolved successively with $C_{out}$ small convolution kernels with the size of $3\times 3\times C_{out}$ for M−1 times and the output feature map $F_{out}$ with the size of $W_{out}\times H_{out}\times C_{out}$ is produced. It is reported that this scheme could not only significantly decrease the trainable parameters and computational cost but also improve the representation learning capability of the convolution layer due to the increasing nonlinearity induced by the activation function of the cascaded 3×3 convolution layers.

**Figure 5:**
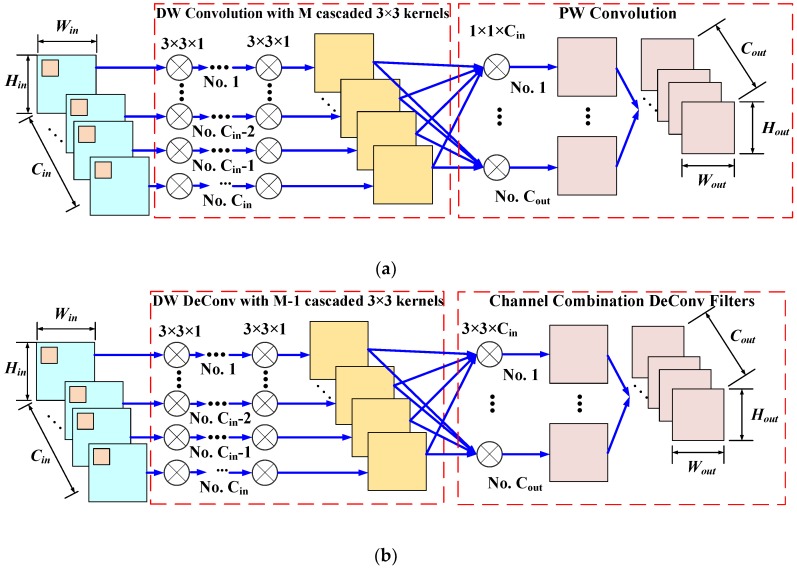
The proposed compact depth-wise separable convolution (CSeConv) and the corresponding compact separable deconvolution (CSeDeConv)layer which combines the depth-wise (DW) separable convolution/deconvolution scheme and the kernel decomposition scheme to reduce the trainable parameters. (**a**) The procedure of the CSeConv layer; (**b**) the details of the CSeDeConv. The size of the input feature map and output feature map in (**a**) and (**b**) are $W_{in}\times H_{in}\times C_{in}$ and $W_{out}\times H_{out}\times C_{out}$, respectively.

Although these schemes significantly decrease the trainable parameters and computational cost, the small benchmark in SAR ATR still limit the application of deeper and complicated models. In this paper, a more compact convolution layer and its deconvolution counterpart are proposed. The proposed layers combine the kernel decomposition scheme and the DW SeConv scheme, thereby requiring a smaller number of trainable parameters as well as introducing more nonlinearity for better representation learning. The proposed compact DW separable convolution (CSeConv) process is depicted in Figure 5a. Similar to the DW SeConv layer, the standard convolution is split into two steps: the DW convolution for separable convolution at the channel level and the PW convolution to combine the filtered features of all channels. Besides, the kernel decomposition scheme is also adopted in the DW convolution step, as each DW convolution can be considered as an input with single-channel convolving with only one kernel. Each large DW kernel is decomposed into a bunch of 3×3 filters, each of which is followed by a nonlinear activation function to provide additional nonlinearity [45]. Accordingly, the trainable parameters can be further decreased by the combined scheme.

Its deconvolution counterpart, i.e., the compact separable deconvolution (CSeDeConv) layer presented in Figure 5b, is devised in the same manner, composed of two steps: the DW separable deconvolution and the channel-level combination DeConv. In the first step, the deconvolution operator was applied to each channel of the $W_{in}\times H_{in}\times C_{in}$ input feature map, $F_{in}$. The kernel decomposition scheme is also employed to split the $N_{k}\times N_{k}$ deconvolution kernel into M−1 concatenated 3×3 filters at the channel level, where M is determined according to (1). Subsequently, $C_{out}$ deconvolution filters with the size of $3\times 3\times C_{in}$ are utilized to combine the output of the DW separable deconvolution step and generate the output feature map $F_{out}$ with the size of $W_{out}\times H_{out}\times C_{out}$. It should be noted that if the stride of either the CSeConv or the CSeDeConv is larger than 1, the convolution/deconvolution operator with the given stride will be implemented in the last channel level combination step.

**Figure 6:**
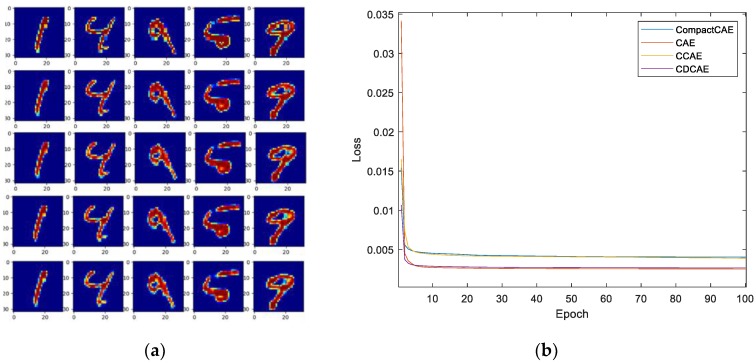
**Validation Experiments with the** mixed national Institute of standards and technology database (MNIST) **dataset**. (**a**) The reconstruction results of the baseline convolution auto-encoder (CAE), the CAE with the compact depth-wise separable convolution (CSeConv) layer (CCAE), the CAE with the compact separable deconvolution (CSeDeConv) layer (CDCAE), and the compact CAE with the CSeConv layer and CSeDeConv layer (CompactCAE) from the top row to the bottom row. (**b**) The training losses of the four models.

To demonstrate the validation of the CSeConv and the CSeDeConv, the mixed national Institute of standards and technology database (MNIST) of handwritten digits was utilized for evaluation. A three-layer CAE model with one standard convolution layer and one deconvolution layer was employed as the baseline model for comparison. Both the convolution layer and the deconvolution layer had four 5×5 filters, and the strides of both the convolution layer and the deconvolution layer were 4. The trainable parameters were initialized with the He initialization [46], while the activation functions of both the convolution and deconvolution layer were ReLU. In the experiment, the convolution and deconvolution layers were replaced by the proposed CSeConv and the CSeDeConv, respectively. Accordingly, four CAEs could be generated for comparison: the baseline CAE, the CAE with the CSeConv layer (CCAE), the CAE with the CSeDeConv layer (CDCAE), and the compact CAE with the CSeConv layer and CSeDeConv layer (CompactCAE). The original images and the reconstructed results of the four CAE models are shown in Figure 6a to illustrate the validation of the proposed layer. Moreover, the training losses of the four models are compared in Figure 6b. As shown in Figure 6, both the reconstruction results and the training losses of the four CAEs were approximately the same which demonstrate the validation of the proposed CSeConv layer and CSeDeConv layer.

A brief analysis of the trainable parameters and computational consumption of various convolution layers are made and compared in Table 1. In our comparison, the size of the input feature map is supposed to be $W_{in}\times H_{in}$ with $C_{in}$ channels, and the size of the convolution kernel is $N_{k}\times N_{k}\times C_{in}$. In order to simplify the analysis, the stride of the convolution is assumed to be 1, and the padding mode of the convolution is set to unify the input and output feature maps in size. Consequently, the size of the output feature map is $W_{in}\times H_{in}$ with $C_{out}$ channels. Besides, the addition of feature aggregation is also ignored as in Reference [36] when the computational consumption with different convolution layers is compared. The number of trainable parameters $K_{param}$, the computation consumption $L_{comp}$, and the ratio of calculation consumption between the improved convolution layer and the standard convolution $R_{opt}=L_{comp}^{Other}/L_{comp}^{Standard}$ are all listed in Table 1. It can easily be found that the number of trainable parameters and the calculation consumption have been effectively reduced compared with the standard convolution and other mainstream convolution layers. Moreover, the reduction of the trainable parameters and the ratio of calculation consumption is only related to the number and size of the convolution kernel.

### 2.3. Multi-Scale Representation Learning with Pyramid Pooling Module and Feature Aggregation Module

#### 2.3.1. Pyramid Pooling Module for Multi-Scale Feature Extraction

In most convolution-based deep networks, the spatial pooling operator is utilized as a crucial element to fuse characteristics of nearby feature bins into a compact representation. The objective of the spatial pooling process is to transform the joint feature representation into a new compressed, more effective one that preserves discriminative information while discarding irrelevant detail, the crux of which is to determine what can benefit the classification performance. Various pooling operators have been devised based on the sum, the average, the maximum, or some other combination rules and achieved significant success in computer vision and SAR ATR tasks [47]. However, most of the spatial pooling operators usually obtain the compact representation at a fixed-size receptive field which is possibly improper to the structure of the intrinsic characteristics and will lead to either information loss with too large of a size or feature dilution with too small of a size. Besides, for targets with a complicated characteristic structure, the fixed-size pooling operators that can only learn features at a fixed scale is also the major cause for incomplete representation learning and the consequent performance degradation.

**Figure 7:**
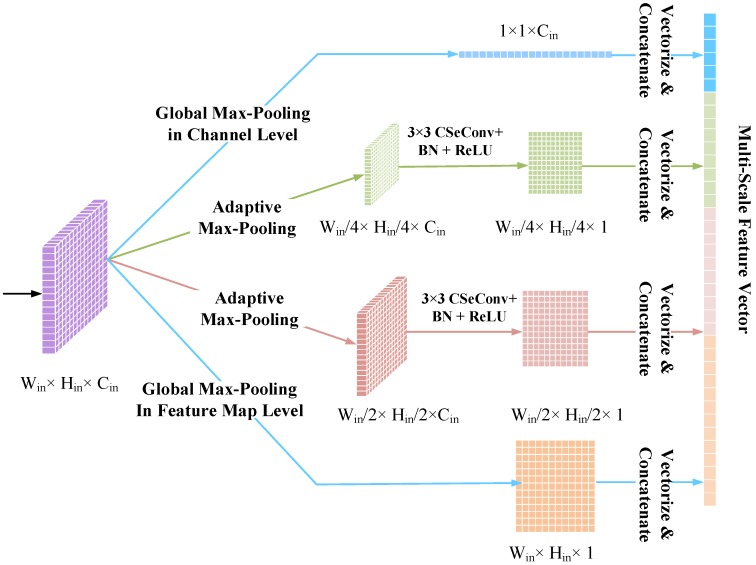
The architecture of the proposed pyramid pooling module (PPM) for multi-scale representation learning at each modality level. Given an input feature map with the size of $W_{in}\times H_{in}\times C_{in}$, the output feature maps of the four sub-branches of the PPM are with the size of $1\times 1\times C_{in}$, $\frac{W_{in}}{4}\times \frac{H_{in}}{4}\times C_{in}$, $\frac{W_{in}}{2}\times \frac{H_{in}}{2}\times C_{in}$, and $W_{in}\times H_{in}\times 1$.

To tackle the problem caused by the fixed-size pooling operators, the pyramid pooling module (PPM) was devised which was first adopted to generate fixed-length representations from inputs with varying sizes for deep visual recognition [48]. The PPM provides an effective way to obtain intrinsic characteristics of complicated targets from the view of multiple scales. In this paper, a modified version of PPM was devised and adopted in the proposed model to obtain a multi-scale representation at each modality level. As depicted in Figure 7, a typical PPM in the proposed model consists of four sub-branches with varying local reception field for pooling to capture the context information of the input feature maps. The first and the last sub-branches are the global max-pooling layer in the channel level and feature map level, respectively. For the two middle sub-branches, each of them consists of an adaptive max-pooling layer and a 3×3 CSeConv layer followed by a BN operator and a ReLU activation function. To be more specific, let us suppose the size of the input feature map is $W_{in}\times H_{in}\times C_{in}$. Accordingly, the size of the output feature maps of the first and the last global max-pooling layers are $1\times 1\times C_{in}$ and $W_{in}\times H_{in}\times 1$, respectively. The size of the output feature maps of the adaptive max-pooling layers in the two middle branches will be $\frac{W_{in}}{2}\times \frac{H_{in}}{2}\times C_{in}$ and $\frac{W_{in}}{4}\times \frac{H_{in}}{4}\times C_{in}$. The following CSeConv layers in each branch is employed to compress the pooled multi-channel feature map into a single-channel feature map, i.e., the sizes of the output feature maps of the CSeConv layers in the two middle sub-branches are $\frac{W_{in}}{2}\times \frac{H_{in}}{2}\times 1$ and $\frac{W_{in}}{4}\times \frac{H_{in}}{4}\times 1$. Finally, the output feature maps of the four sub-branches are converted to a single column vector and concatenated to construct the representation at the current modality level. It should be noted that if the size of the input feature map is too small to obtain the feature maps in the overall four sub-branches, part of the branches can be removed from the typical PPM and the corresponding feature maps can be neglected according to the image size.

#### 2.3.2. Feature Aggregation Module (FAM) for Feature Map Reconstruction

**Figure 8:**
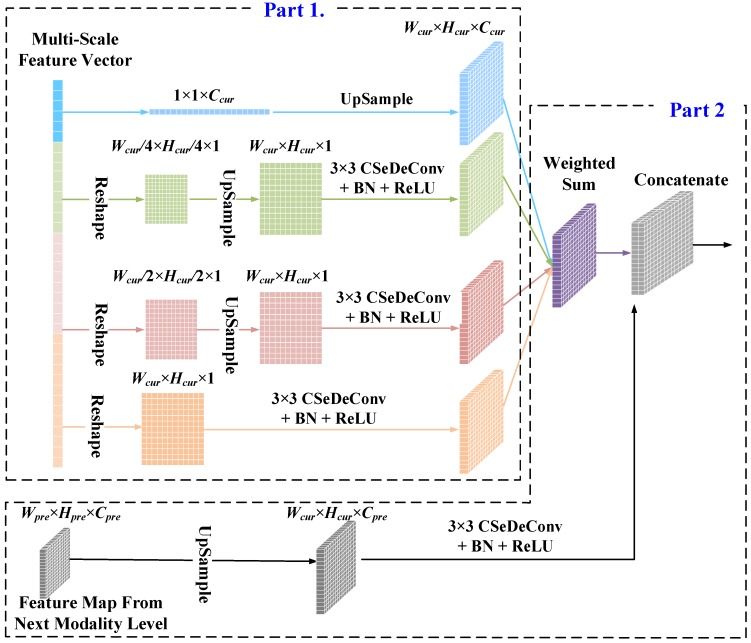
The architecture of the proposed feature aggregation module (FAM) for merging multi-scale representation of different modality levels which consists of two parts. The first part of the proposed module combines multi-scale feature maps and generates a new feature map at the current modality level. The input feature vector with the size of $\frac{21}{16}W_{cur}\times H_{cur}+C_{cur}$ is decomposed and reshaped to produces the feature maps in the four sub-branches with the size of $1\times 1\times C_{cur}$, $\frac{W_{cur}}{2}\times \frac{H_{cur}}{2}\times 1$, $\frac{W_{cur}}{4}\times \frac{H_{cur}}{4}\times 1$ and $W_{cur}\times H_{cur}\times 1$ Subsequently, the upsampling operator and the following smoothing operator are applied to each sub-branch. The output feature maps of the four sub-branches are weighted and added to generate the feature map of the first part with the size of $W_{cur}\times H_{cur}\times C_{cur}$. The second part of the FAM merges the feature map with the size of $W_{pre}\times H_{pre}\times C_{pre}$ from the coarser modality level with the combined feature map with the size $W_{cur}\times H_{cur}\times C_{cur}$ at the current modality level. The input feature map at the coarser level is upsampled and smoothed to generate the upsampled feature map of the coarser level with the size of $W_{cur}\times H_{cur}\times C_{pre}$ The upsampled feature map is concatenated with the feature map at the current modality level, generating the new feature map with the size of $W_{cur}\times H_{cur}\times \left(C_{pre}+C_{cur}\right)$.

The utilization of our PPMs in the encoder stage allows the model to learn multi-scale representations from the input SAR image at different modality levels. However, a new problem that deserves to be solved is how to seamlessly merge the feature maps from PPMs at different modality levels and obtain the reconstructed image that is the essential objective of an AE model. To this end, a series of FAMs are developed each of which contains two parts as illustrated in Figure 8.

In the first part, the feature maps at different scales of the PPM are combined to produce a new feature map at the current modality level. The multi-scale feature vector at one modality level obtained by a PPM is first decomposed and reshaped into feature maps of different scales according to the PPM at the same modality level. Subsequently, the feature maps of different scales are processed in separate sub-branches. For two middle sub-branches, an upsampling operator and a smooth operator consisting of a 3×3 CSeDeConv layer, a BN operator and a ReLU activation function are executed. For the first and the last sub-branches, only the upsampling operator and the smooth operator are applied, respectively. Finally, the processed feature maps of different scales are weighted and summed to generate the feature map of the current modality level. In the second part, the feature map from the coarser level is merged with the combined multi-scale feature map at the current level. The upsampling process followed by a 3×3 CSeDeConv layer, a BN operator and a ReLU activation function is applied to obtain the feature map of a coarser level that is the same size as the feature map at the current level. Subsequently, feature maps from different levels are concatenated together to generate the merged feature map.

To be more specific, in the first step, suppose the input feature vector at the current modality level has the size of $\frac{21}{16}W_{cur}\times H_{cur}+C_{cur}$. The input multi-scale feature vector will be decomposed and reshaped the feature maps with the size of $1\times 1\times C_{cur}$, $\frac{W_{cur}}{2}\times \frac{H_{cur}}{2}\times 1$, $\frac{W_{cur}}{4}\times \frac{H_{cur}}{4}\times 1$, and $W_{cur}\times H_{cur}\times 1$, respectively. Subsequently, the upsampling operator and the following smoothing operator are applied. Accordingly, the output feature maps of the four sub-branches will have the same size of $W_{cur}\times H_{cur}\times C_{cur}$. Finally, the four feature maps are weighted and added to generate the feature map of the current modality level with the size of $W_{cur}\times H_{cur}\times C_{cur}$. In the second step, let the input feature map at the coarser level have the size of $W_{pre}\times H_{pre}\times C_{pre}$ with $W_{pre}\times H_{pre}$ being the spatial size of the input feature map and $C_{pre}$ being the number of channels at the coarser level. The feature map from the coarser level is upsampled and smoothed by a 3×3 CSeDeConv layer, a BN operator, and a ReLU activation function. Finally, the upsampled feature map of the coarser level with the size of $W_{cur}\times H_{cur}\times C_{pre}$ is concatenated with the feature map at the current modality level, generating the new feature map with the size of $W_{cur}\times H_{cur}\times \left(C_{pre}+C_{cur}\right)$.

### 2.4. Loss Function Based on the Modified Reconstruction Loss and Speckle Filtering Restriction

Typically, an AE-based model provides a symmetrical frame on learning latent representation of candidate targets by mapping the inputs into a low dimensional feature space at the encoder stage and approximately reconstruct the inputs from the learned features at the decoder stage. The objective of the AE-based model is to minimize the loss function that measures the distortion between the inputs and the outputs to guarantee that the mapping process preserves the information of the inputs. The commonly used loss functions, such as the MSE, cross-entropy, and the Minkowski distance in the field of deep learning, concern the total bias of pixel values or distributions and neglect the structural information of the candidate targets, leading to performance degradation in SAR ATR. Therefore, the SSIM loss function [49] is employed in the proposed model which simultaneously compares the similarity of two images over the structure, the luminance, and the contrast, gaining significant success in the computer vision domain. Suppose the input image of an AE-based model is x and the output of the model is $\hat{x}$, the SSIM loss function can be:

(2) $$L_{SSIM}\left(x,\hat{x}\right)=E\left(\frac{\left(2\mu_{x}\mu_{\hat{x}}+c_{1}\right)\left(2\sigma_{x\hat{x}}+c_{2}\right)}{\left(\mu_{x}^{2}+\mu_{\hat{x}}^{2}+c_{1}\right)\left(\sigma_{x}^{2}+\sigma_{\hat{x}}^{2}+c_{2}\right)}\right),$$

where $\mu_{x}$ and $\mu_{\hat{x}}$ are the local average in a 11×11 sliding window of x and $\hat{x}$, respectively; $\sigma_{x}^{2}$ and $\sigma_{\hat{x}}^{2}$ are the local variance in a 11×11 sliding window of x and $\hat{x}$, respectively; $\sigma_{x\hat{x}}$ is the correlation coefficient in a 11×11 sliding window; $c_{1}={\left(K_{1}L\right)}^{2}$ and $c_{2}={\left(K_{2}L\right)}^{2}$ are two constants with $K_{1}=0.01$ and $K_{2}=0.03$; L is the dynamic range of the pixel values (1.0 for normalized SAR images); E(·) is the expectation operator. While calculating the SSIM loss of two images, the sliding window will be moved pixel by pixel over the entire image. At each step, the local statistics and the local SSIM loss are computed in the window. Finally, the SSIM of the entire image is computed by averaging the local SSIM of each step.

Another problem is that in most conditions there is serious speckle in the target patches which not only have little information about the target but affect the ATR capability of the learned features. To alleviate their influence, the reconstruction loss is modified by measuring the distortion between the outputs and the speckle filtered images instead of the original input data. Consequently, the model will be forced to learn the characteristics of the targets rather than the background clutter, and the prior knowledge of speckle suppression can be embedded in the MSCAE during the model training procedure. In the proposed model, the ILSF is employed to generate the speckle suppressed image due to the fact of its excellent capability in maintaining detailed structures, strongly reflecting and scattering targets, and smoothing undesired background clutter [50]. Moreover, an additional restriction is devised to guarantee that the speckle suppression process is taken place in the encoder stage and little information on the speckle will be learned. The restriction is implemented by comparing the difference between the features learned from the original inputs and those learned from the speckle suppressed images. Accordingly, the loss function of the proposed model is

(3) $$L_{MSCAE}=\frac{1}{N}\sum_{i=1}^{N}L_{SSIM}\left({\hat{x}}_{i},x_{i}^{ILSF}\right)+\alpha \sum_{j=1}^{C}||h_{ij}-h_{ij}^{ILSF}{\left|\right|}_{2}$$

where $D_{train}={\left\{x_{i}\right\}}_{i=1}^{N}$ is the training dataset with $x_{i}$ being the ith target patch and N being the number of samples; ${\hat{x}}_{i}$ and $x_{i}^{ILSF}$ are the output image of the proposed model and the speckle suppressed version of $x_{i}$, respectively; $||\cdot {\left|\right|}_{2}$ is the l−2 norm; $h_{ij}$ and $h_{ij}^{ILSF}$ are the encoded feature vectors of $x_{i}$ and $x_{i}^{ILSF}$ at scale j, respectively; C is the number of modality levels; α is the coefficient of the speckle suppression restriction, which can be 0.01/C in most SAR ATR tasks.

## 3. Experiments and Discussion

### 3.1. Experimental Data Sets

**Figure 9:**
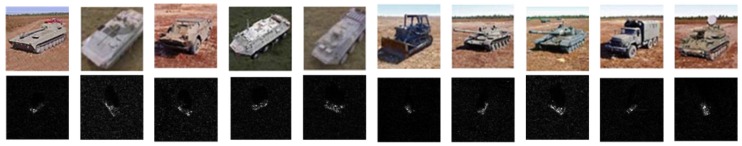
Photographs (the first row) and SAR imagery examples (the second row) of the moving and stationary target acquisition and recognition (MSTAR) dataset for model evaluation. From left to right, the types of vehicles are 2S1, BMP-2, BRDM-2, BTR-60, BTR-70, D7, T-62, T-72, ZIL-131, and ZSU-234.

In this study, the representation learning capability of the proposed model was evaluated by the MSTAR dataset [42] which is jointly sponsored by the US Defense Advanced Research Projects Agency (DARPA) and Air Force Research Laboratory (AFRL). There were a total of ten distinctive types of vehicles in the dataset as shown in Figure 9, including the armored personnel carrier BMP-2, BRDM-2, BTR-60, and BTR-70; the tank T-62 and T-72; the rocket launcher 2S1; the air defense unit ZSU-234; the truck ZIL-131; and the bulldozer D7. The images were collected by an X-band SAR in spotlight mode with the resolution of 0.3 m×0.3 m and split into tens of thousands of small patches centered on the candidate targets and surrounded by varying background clutter. These small patches provide full-aspect coverage from 0° to 360° and different views at various depression angles for each type of the ten vehicles. Detailed information including the type, the serial number (Serial No.), the depression angle, and the number of samples are all listed in Table 2.

In order to ensure comprehensive access to the performance, the proposed MSCAE was tested under standard operating condition (SOC) and various extended operating conditions (EOCs) including substantial variations in the signal-to-noise ratio (SNR), resolution, and version. In our experiments, the proposed model was first validated on three similar targets, namely, BMP-2, BTR-70, and T-72, to validate its performance under SOC and version variants. Subsequently, validation of the SSIM loss, the PPM, the CseConv, and CSeDeConv and the speckle suppression scheme are all discussed based on the three-target dataset. Experiments on 10 class MSTAR data were also conducted to evaluate the performance under the extension of the target type. Finally, the robustness of the proposed model under various conditions, including noise corruption and resolution variance, was also evaluated with the ten-target dataset. For both the three-target dataset and the ten-target dataset, the patches acquired at the 17° depression angle were utilized as training samples, while those obtained at the 15° depression angle constructed the test set. Similar to the experimental setting in References [30,31], only the data from BMP2-9563 and T72-132 were used, as the samples of the BMP-2 and T-72 were used to construct the training dataset. But in the test dataset, images of all serial numbers (i.e., version variants) were used to test the performance of the proposed method.

### 3.2. Experiment Configuration

#### 3.2.1. Data Preprocessing

In most deep networks, including the proposed model, the size of the input images is required to be the same. Meanwhile, the size of target patches in the MSTAR dataset can vary from 128×128 to 158×158. Consequently, the input target patches should be resized to the same shape, which is 128×128 in this study, before being used for model training and performance validation. In this study, the image crop processing based on the centroid of the target region is adopted. The ILSF is firstly applied to suppress the speckle and background clutter in the small patches. Subsequently, a two-parameter constant false alarm rate (CFAR) detector is executed to obtain the target region of each patch. The centroid is calculated by averaging the coordinates of the target pixels weighted by their pixel values. Finally, only the 128×128 region surrounding the centroid will remain, while other regions will be removed.

Another preprocessing step is the normalization process. It can be found that in many target patches, the intensity of targets seriously varies which possibly conceals the differences among targets and, thus, affect the performance of the learned features. Accordingly, intensity normalization was adopted to alleviate the amplitude variation in target patches, mapping the pixel intensities onto the range [0,1]. Except for image resizing and normalization, no other preprocessing, such as data augmentation (DA) or target segmentation, were applied.

#### 3.2.2. Model Configuration and Experiment Design

In our experiments, an MSCAE with four modality levels was utilized to obtain multi-scale representations of the MSTAR data. The model parameters and the fan-ins and fan-outs of each level are listed in Table 3. As depicted in the table, there were some changes in the third and fourth levels. In the third level, the size of the feature map was downsampled from 16×16×16 to 4×4×32 after the 2×2 max-pooling and the 5×5 CseConv with a stride of 2. While feeding the feature map to the PPM block, the output feature maps of the four sub-branches of the typical PPM should have the size of 4×4×1, 2×2×32, 1×1×32 and 1×1×32, respectively. The outputs of the third and the fourth sub-branch were the same, bringing in redundant information and had little contribution to the target discrimination. Accordingly, the fourth sub-branch, which provides a feature map with global max-pooling in channel level, was removed, and the corresponding feature map was neglected. The FAM in the same modality level was also changed by removing the corresponding sub-branches while combining the feature maps obtained by the PPM. In the fourth modality level, since the size of the input feature map was smaller than 5×5, the optional CseConv layer was removed, and only the 2×2 max-pooling layer was applied before drawing the feature vector of the coarsest level.

All the convolution kernels of the MSCAE were also initialized with the He initialization [46]. After initialization, the model was trained with the preprocessed training dataset, and the Adam optimizer [51] was utilized to optimize the model with an initial learning rate of 0.001. The exponential decay rates $\beta_{1}$ and $\beta_{2}$ for the moment estimates were 0.9 and 0.999, respectively. The batch of the training samples was 32. The maximum number of iterations was 500, and the early-stopping scheme was enabled to terminate the training if the improvement of the training loss was less than the threshold. In our experiments, the nonlinear SVM (NSVM) was employed for classification after extracting features from the target patches. Moreover, to avoid fluctuations in the results caused by random steps in the model initialization and optimization, each experiment was repeated ten times, and the average of the results were utilized for performance evaluation.

Experiments were carried out in a 64 bit Windows 10 system. The proposed model was mainly built on the Google Tensorflow v1.5.0 deep learning library in the Python development environment PyCharm. The hardware platform was a specially adapted DELL T5810 workstation with an Intel Xeon E5-1607 v3 @ 3.10 GHz CPU, 32 GB DDR4 RAM and an NVIDIA K40c (12G memory) GPU with CUDA8.0 accelerating calculation.

### 3.3. Evaluation on Three-target Classification

The average results of the ten experiments with the three-target dataset are depicted in Table 4. The performance was measured by the probability of correct classification ($P_{cc}$) which is calculated through the number of targets recognized correctly divided by the number of all the targets. The results with and without version variants are listed in the sixth and fifth columns of the table, respectively. A comparison experiment was also conducted to demonstrate the performance improvement induced by the multi-scale feature learning architecture. Classification rates with various feature combinations are evaluated and compared.

As shown in Table 4, for the experiment without version variants (i.e., under SOC), the proposed model had the highest accuracy (i.e., 99.73%) when features from all modality levels were utilized. Meanwhile, in the case with variants only, the accuracy of the proposed model with various feature combinations suffered a slight degradation due to the differences in local structure and small equipment of varied serial numbers. However, the accuracy obtained by the model with features from all levels was still higher than 98%, indicating a good generalization performance. The average accuracies of these methods with all the test data are listed in the seventh column of Table 4. It can be found that when features from multiple scales are combined, the average $P_{cc}$ of 99.14% is competitive to the state-of-the-art results provided by the supervised neural networks. The major reason is that the proposed two-level multi-scale feature extraction structure guarantees that the MSCAE can learn the high-level abstracted properties while preserving the detailed information that is neglected by most DL networks. Besides, the specifically designed objective function with speckle suppression and SSIM can diminish the influence of serious speckle in SAR images and take full advantage of the target structure caused by the backscattering. In addition, the proposed compact convolution and deconvolution processes greatly decrease the number of trainable parameters and introduce more nonlinearity that slightly benefits the model capability.

**Figure 10:**
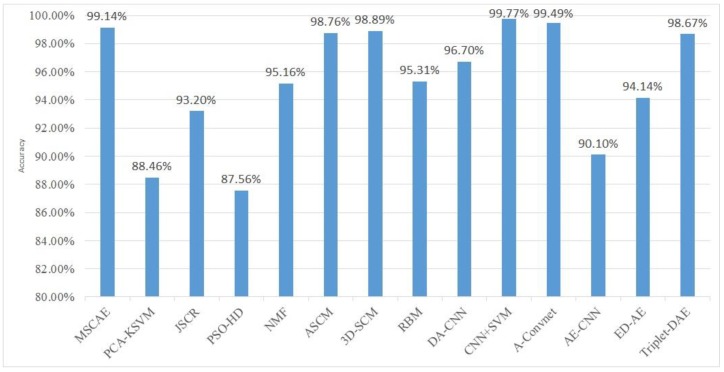
Performance comparison with baseline handcrafted feature extraction methods and deep representation models via the three-target dataset.

Other features extraction methods were also compared with the proposed method for further evaluation including the baseline handcrafted methods and the DL networks which were obtained from the state-of-the-art results. The baseline handcrafted methods include the PCA-kernel SVM (PCA-KSVM) [52], the joint sparse representation based method (JSRC) [53], the particle swarm optimization with Hausdorff distance (PSO-HD) [54], the non-negative matrix factorization (NMF) method [55], the attributed scattering center matching method (ASCM) [7], and the 3D scattering center model reconstruction method (3D-SCM) [5]. Among these methods, the PCA-KSVM method employs the nonlinear PCA to extract discriminative feature and, subsequently, feeds the features into the SVM classifier. The JSRC method exploits the inter-correlations among the multiple views using joint sparse representation over a training dictionary. The PSO-HD is a pattern matching method that minimizes the Hausdorff distance over rigid transformations. The NMF method utilized the NMF with an L_1/2_ norm constraint to extract features in SAR images. The ASCM and the 3D-SCM were devised based on the backscattering model of the SAR image and achieved state-of-the-art results in SAR ATR. The ASCM proposed a SAR ATR method, where the ASCs were utilized for target reconstruction and similarity measurement. In the 3D-SCM method, the 3D scattering center model, established offline from the CAD model of the target, was employed to predict the 2D scattering centers for template matching. The DL networks for comparison were composed of the restricted RBM (RRBM) [56], the CNN with DA (DA-CNN) [57] and additional data generated by image processing methods, the CNN with SVM (CNN+SVM) [37], the A-Convnet [57] that replaced the fully connected layers with a convolution layer in a CNN, the sparse AE pre-trained CNN (AE-CNN) [58] where the convolution kernel was trained on randomly sampled image patches using unsupervised sparse auto-encoder, the ED-AE [30], and the Triplet-DAE [31]. Among these methods, the CNN with SVM and the A-Convnet were implemented in our codes with Python. In our implementation, preprocessing included image cropping, speckle filtering with ILSF, and normalization was applied to these methods. Besides, the additional DA scheme was also executed to generate sufficient training samples for the CNN with SVM model and the A-Convnet according to References [37,38]. The configurations of the CNN with SVM and the A-Convnet were determined according to References [37,38]. The accuracies of all the methods are shown in Figure 10. The features learned by the proposed method had a better classification capability than most handcrafted methods, even comparing them with ASCM and 3D-SCM which achieved state-of-the-art results for handcrafted features, because of the multi-scale feature learning scheme and the specifically designed objective function. Comparison with deep networks, including the DA-CNN, the RRBM, the AE-CNN, and the ED-AE, also indicates that the proposed model outperformed most of the DL models which have specialized restrictions for finding discriminative features. Even compared with the CNN+SVM and the A-Convnet that achieved state-of-the-art results, the proposed method obtained a comparable result.

### 3.4. Validation of the Model Component

**Figure 11:**
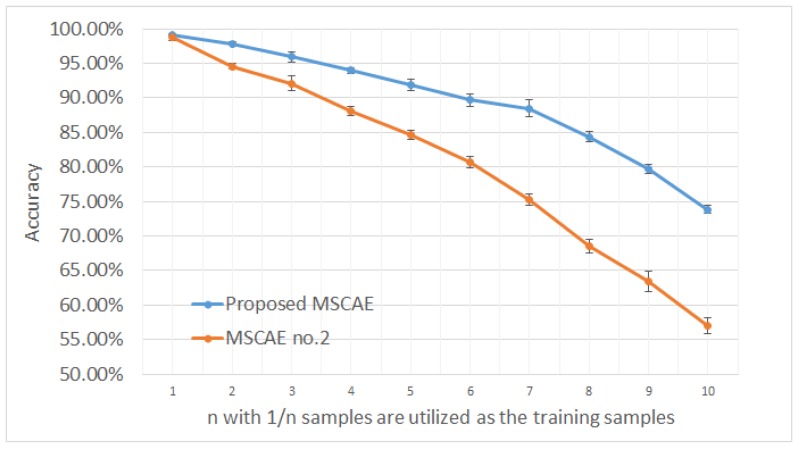
Validation of the proposed model with a small dataset when 1/*n* samples were randomly selected to train the model.

In order to investigate the contribution of each proposed component in the MSCAE, including the PPM, the CSeConv, the SSIM measurement, the ILSF, and the speckle suppression restriction, validation experiments were conducted. Each component was removed from the MSCAE to reveal the performance improvement induced by it. Accordingly, we obtained five models for performance validation, marked as MSCAE no. 1 to no. 5 in Table 5. In model no. 1, the PPMs and the corresponding FAMs at each modality were removed from the MSCAE, and the multi-scale features were only generated by the U-shaped architecture. In model no. 2, the CSeConv and the corresponding CSeDeConv layers were all replaced by the standard convolution layers. In model no. 3, the SSIM measurement was replaced by the MSE loss. In model no. 4, all the data flows and restrictions that related to the ILSF were removed from the MSCAE model, while in model no. 5 only the restriction term in the objective function was removed. The three-target MSTAR dataset was utilized to evaluate their performance and each experiment was conducted ten times. The average accuracy of the five models and the proposed MSCAE models are listed in Table 5. As shown in the table, the PPM and the SSIM measurement contributed the most to improving the accuracy, 2.85% and 1.83% respectively, while CSeConv and the CSeDeConv only improved the accuracy approximately 0.41%. However, the proposed CSeConv and CSeDeConv can remarkably reduce the number of trainable parameters in the proposed model that can greatly benefit its performance with a small training dataset. To further illustrate their contribution, an experiment which evaluated the model performance with a limited training sample was conducted by randomly removing a part of the sample in the training set. In this experiment, only 1/n images were randomly selected from the dataset as training samples with n varying from one to ten. The average accuracies and their standard deviations with the proposed MSCAE and model no. 2 are presented in Figure 11. As shown in the figure, the proposed model achieved an accuracy higher than 90% when only 20% of the sample was utilized to train the model, while the *P_cc_* of the MSCAE no. 2 that had much more trainable parameters than the proposed model fell below 85%. Moreover, when the size of the training dataset was only 1/10 of the original one, the *P_cc_* of the MSCAE no. 2 fell below 60% and the proposed MSCAE still had an accuracy higher than 70%.

### 3.5. Evaluation on Ten-Target Classification

The average results of the ten experiments with the ten-target MSTAR dataset are depicted in Table 6. It can be found that by combining the features of all the four levels, the classification accuracy obtained an improvement that increased from 98.5% to 98.9%, in comparison with the highest $P_{cc}$ achieved by the feature vector that combined the learned representations of the first three modality levels. Many other feature extraction methods and representation learning models were also compared with the MSCAE for further evaluation, including the baseline handcrafted features, the unsupervised DL models, and the supervised models. The baseline handcrafted features for evaluation includes the NMF method [55], the sparse representation of monogenic signal via Riemannian manifolds (SRRMs) [59], the weighted multi-task kernel sparse representation (WMTKSR) [60], and the ASCM [7]. Among these methods, the SRRM utilizes the covariance descriptor of the monogenic signal as the features, and classifies the targets with the Riemannian manifold embedded in an implicit reproduction of the kernel Hilbert space (RKHS). The WMTKSR maps the multi-scale monogenic features into a high-dimensional kernel feature space using the nonlinear mapping associated with a kernel function, and the classification process is formulated as a joint covariate selection problem across a group of related tasks. The unsupervised DL models comprise the multi-discriminator generative adversarial network (MGAN-CNN) that generates unlabeled images with GAN and sets them as the input of CNN together with original labeled images [61], the feature fusion SAE (FFAE) [15] that extracts 23 baseline features and three-patch local binary pattern (TPLBP) features and, subsequently, feeds them into an SAE for feature fusion and the variational AE based on residual network (ResVAE) [22]. The supervised models for performance evaluation are the ED-AE [30], the Triplet-DAE [31], the CNN with SVM [37], the A-Convnet [38], the ESENet that based on a new enhanced squeeze and excitation (enhanced-SE) module [35], and the hierarchical fusion of CNN and ASC (ASC-CNN) that provide a complicated scheme to fuse the decision of the ASC model and the CNN [39]. Among these methods, the CNN with SVM and the A-Convnet are implemented in our codes with Python. In our implementation, preprocessing included image cropping, speckle filtering with ILSF, and normalization was applied to the two methods. Besides, an additional DA scheme was also executed to generate sufficient training samples for the CNN with an SVM model and the A-Convnet according to References [37,38]. Therefore, both the results of the two models with and without DA processes were compared in our experiment to make a comprehensive and equal analysis. The configurations of the CNN with SVM and the A-Convnet were determined according to References [37,38]. In the experiments, each of the CNN with SVM and the A-Convnet was executed and tested ten times, and the average classification accuracy was utilized for performance evaluation.

**Figure 12:**
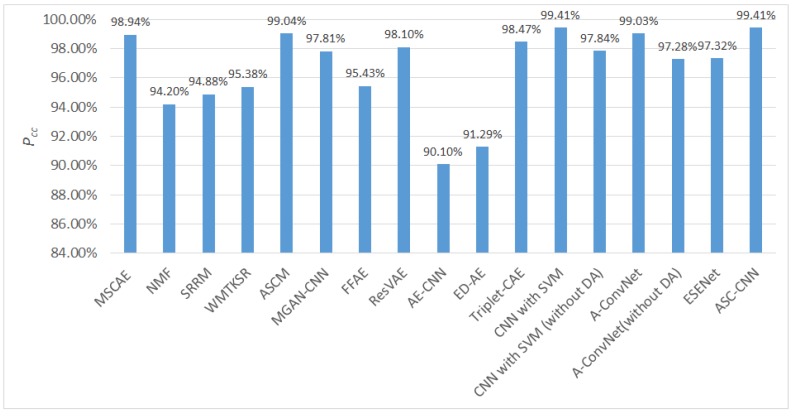
Performance comparison on the ten-target dataset with different handcrafted feature extraction methods and deep representation models.

The classification results of all the methods are depicted in Figure 12. It can easily be found that the classification accuracy of the proposed method was much higher than most of the traditional handcrafted features. Although the accuracy of the proposed model was a bit lower than the ASCM feature that achieved state-of-the-art results with the scattering center model, the result obtained by the proposed model was still comparable and can adaptively extract features without manual intervention. Compared with most of the deep representation learning methods (e.g., the ED-AE, the Triple-DAE, the MGAN-CNN, and the ESENet), the proposed model also yielded much better performance. In comparison with the CNN with SVM, the A-Convnet and the ASC-CNN that achieved state-of-the-art results, the proposed MSCAE was also competitive. The results achieved by the CNN with SVM and the A-Convnet without DA preprocess demonstrated that their high classification rates mainly relied on the DA operations. Although their DA processes did improve the performance, they induced certain problems including bringing in man-made uncertainty and unstable performance, amplifying the sampling biases in the original dataset, and high computational complexity. The results obtained by the ASC-CNN devised a complex decision fusion strategy to improve the accuracy obtained by the ASC and the CNN separately. Although the performance of the proposed model was better than the proposed model, it requires a complicated process to extract the ASC features and higher computational complexity.

### 3.6. Classification Experiment with Noise Corruption

**Figure 13:**
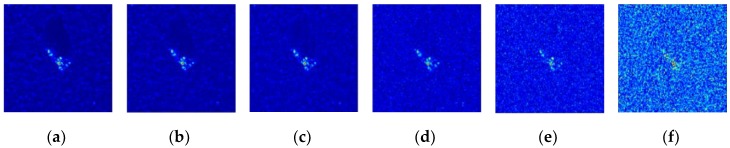
The noise interrupted images with different signal-to-noise ratios (SNRs). (**a**) The original image, (**b**) the SNR at 10 dB, (**c**) the SNR at 5 dB, (**d**) the SNR at 0 dB, (**e**) the SNR at −5 dB, and (**f**) the SNR at −10 dB.

An important characteristic of SAR data is that serious noise can often be observed in the images, which is a major factor causing performance deterioration in SAR ATR. Accordingly, to demonstrate the robustness of the proposed model, the SAR images corrupted by different levels of SNRs were simulated to evaluate the model’s robustness to noise. The original MSTAR images that had an SNR over 30 dB were considered as noise-free sources. To obtain the noise-contaminated images, the original MSTAR patches were first transformed into the frequency-aspect domain with the 2D inverse discrete Fourier transform (IDFT), and different levels of additive complex Gaussian noises were added to the transformed images with the SNR defined in Equation (4) in accordance with Reference [5].

(4) $$NR\left(dB\right)=10log_{10}\frac{\sum_{u=0}^{U-1}\sum_{v=0}^{V-1}{\left|f\left(u,v\right)\right|}^{2}}{HW\sigma^{2}}$$

where f(h,w) denotes the complex RCS computed by the EM code; $\sigma^{2}$ is the variance of the complex noise. By transforming the noisy RCS into the image domain using the same imaging process, the noise-contaminated images can be generated for experimental evaluation. Figure 13 presents some contaminated images with different SNRs.

**Figure 14:**
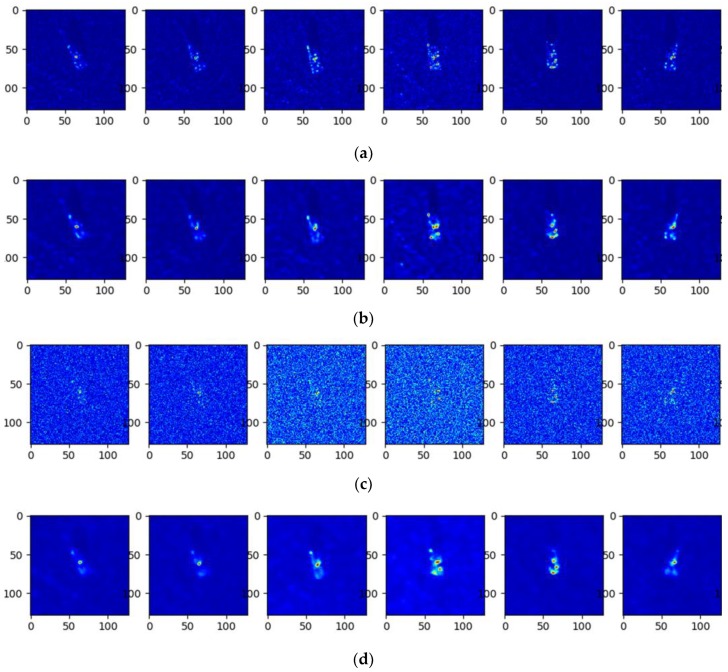
The input images and reconstruction results of the trained model at the noise levels of 10 dB SNR and −10 dB SNR. (**a**) Input images at a noise level of 10 dB SNR, (**b**) the reconstructed results of (**a**), (**c**) the input images at a noise level of -10dB SNR, (**d**) the reconstructed results of (**c**).

**Figure 15:**
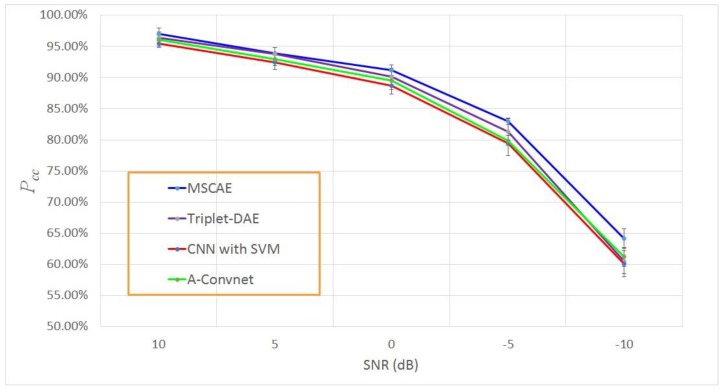
Classification results at different SNR levels.

Some input images and the corresponding reconstruction results at 10 dB and −10 dB SNR are presented in Figure 14 for comparison. Although many inputs at −10 dB SNR were seriously contaminated such that the targets in the patches can merely be observed, the output images of the trained model successfully reconstructed the major parts of the targets, demonstrating the excellent noise suppression capability of the proposed model. The average classification results and the corresponding standard deviations with noise-contaminated data under different SNRs are shown in Figure 15. The average experimental results with other methods are also presented in the figure including the Triplet-CAE, the CNN with SVM, and the A-Convnet. With the decreasing SNR of the input images, the classification accuracy of all the models suffers different degrees of deterioration and the highest was obtained by the proposed model at nearly every SNR level. When the SNR was higher than 0 dB such that the geometric and scattering characteristics were not seriously interrupted by the noise, each model reported a classification rate higher than 85%, and the proposed model achieved the highest accuracy. Even when the noise level was −10 dB such that most of the targets were concealed in the noise, as presented in Figure 13 and Figure 14, the classification rate of the MSCAE still yielded a better performance than the other reference models in Figure 15, demonstrating the robustness of the proposed model under serious noise interruption.

### 3.7. Classification Experiment with Resolution Variance

The proposed model was subsequently evaluated concerning resolution variance. Theoretically, the range resolution and azimuth resolution of the SAR imagery was determined by the bandwidth of the transmitted wave and the synthetic aperture angle. However, due to the instability of the radars, the actual resolution of the measured SAR images would fluctuate around the theoretical values. Meanwhile, it was infeasible to train and maintain models at every possible resolution. Consequently, the robustness of resolution variation is also an important factor for model performance evaluation. Because the resolution of all target patches in the MSTAR dataset was 0.3 m×0.3 m, the target patches with varied resolution should be simulated from the original images in the dataset. The spatial SAR images were converted into the frequency-aspect domain by the 2D-IDFT, and the sub-band was extracted. The sub-band data were subsequently resampled by zero-padding in the frequency domain and turned back to the spatial domain.

**Figure 16:**
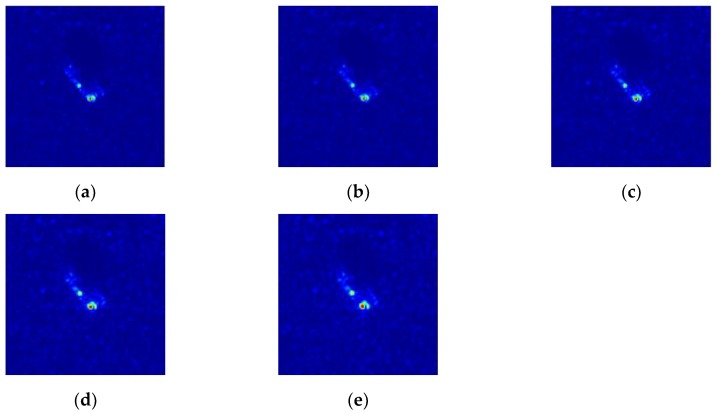
MSTAR data at different resolution. (**a**) 0.3 m × 0.3 m, (**b**) 0.4 m × 0.4 m, (**c**) 0.5 m × 0.5 m, (**d**) 0.6 m × 0.6 m, (**e**) 0.7 m × 0.7 m.

**Figure 17:**
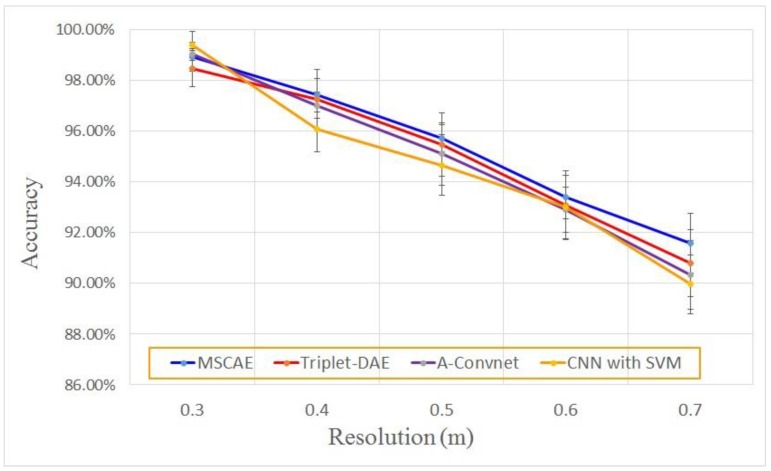
Classification results at different resolutions.

In the evaluation experiment, the resolution of the simulated data varied from 0.3 m×0.3 m to 0.7 m×0.7 m, and some images at different resolutions are presented in Figure 16. Similar to the configuration of the noise interruption experiment, the classification results of the proposed model are compared with the three reference models including the Triplet-DAE, the CNN with SVM, and the A-Convnet. At each resolution level, the experiment of each model was executed ten times to alleviate the influence of randomness caused by the model initialization and optimization. The average experimental results of each model are plotted in Figure 17. As shown in the figure, limited resolution deterioration did not seriously affect the performance of all the models. Even when the resolution was 0.6 m×0.6 m, their average accuracy was still higher than 90%. However, the proposed model still gained the highest classification rate in comparison with the reference models at almost all the resolutions, illustrating its robustness under the extended operation condition of resolution variance.

## 4. Conclusions and Future Work

In this paper, an unsupervised representation learning model was proposed, providing an effective way to learn the multi-scale representation of targets in SAR images via its U-shaped architecture, the CSeConv and the PPM blocks, and the modified loss function based on the SSIM and the restriction of speckle suppression. The major contributions of our work include:

(1) A proposed unsupervised multi-scale representation learning framework for feature extraction in SAR ATR. The utilization of the U-shaped multi-scale architecture and the PPM blocks simultaneously obtained abstract features and local detailed characteristics of targets, boosting the representational power of the proposed model;
(2) An objective function composed of a modified reconstruction loss and a speckle suppression restriction. The reconstruction loss based on SSIM and ILSF forces the MSCAE to learn adaptive speckle suppression capability, while the restriction guarantees that the speckle filtering procedure was implemented in the feature learning step;
(3) The CSeConv and the CSeDeConv decreased the trainable parameters and calculation consumption, avoiding overfitting caused by insufficient samples. Moreover, they introduced more nonlinearity and slightly improved the performance of the MSCAE.

The MSTAR dataset was utilized to evaluate the performance of the proposed model. The proposed method was tested under both standard operating conditions and several extended operating conditions with both the three-target dataset and the ten-target dataset including the version variants, the noise corruption, and the resolution variance. Evaluation experiments demonstrated that the proposed method outperformed most of the conventional and deep learning algorithms and achieved comparable accuracy to the state-of-the-art results without any supervised information.

## Figures and Tables

**Table 1:** Analysis of the trainable parameters and computational consumption of the proposed convolution layer and some mainstream convolution layers.

Layer Name	In	Out	Kernel Size			$K_{param}$	$L_{comp}$	$R_{opt}$
			**Ordinary**	**DW**	**PW**			
Standard	$W_{in}\times H_{in}\times C_{in}$	$W_{in}\times H_{in}\times C_{out}$	$N_{k}\times N_{k}\times C_{in}$	/	/	$C_{out}\times N_{k}\times N_{k}\times C_{in}$	$W_{in}\times H_{in}\times N_{k}\times N_{k}\times C_{in}\times C_{out}$	1
DW SeConv			/	$N_{k}\times N_{k}\times 1$	$1\times 1\times C_{in}$	$N_{k}\times N_{k}\times C_{in}+C_{in}\times C_{out}$	W_in×H_in×N_k×N_k×C_in+W_in×H_in×C_in×C_ou	$\frac{1}{C_{out}}+\frac{1}{N_{k}^{2}}$
DeCConv			1st: $3\times 3\times C_{in}$; Other:$3\times 3\times C_{out}$	/	/	$3\times 3\times C_{in}\times C_{out}+3\times 3\times C_{out}\times C_{out}\times \left(N_{k}-3\right)/2$	W_in×H_in×3×3×C_in×C_out+W_in×H_in×3×3×C_out×C_out×((N_k−3))/2	$\frac{9}{N_{k}^{2}}+\frac{9C_{out}\left(N_{k}-3\right)}{2C_{in}\times N_{k}^{2}}$
CSeConv			/	3×3×1	$1\times 1\times C_{in}$	$3\times 3\times C_{in}\times \left(N_{k}-1\right)/2+1\times 1\times C_{in}\times C_{out}$	W_in×H_in×3×3×C_in×((N_k−1))/2+W_in×H_in×C_in×C_out	$\frac{9\times \left(N_{k}-1\right)}{2N_{k}^{2}\times C_{out}}+\frac{1}{N_{k}^{2}}$
CSeDeConv			/	3×3×1	$3\times 3\times C_{in}$	$3\times 3\times C_{in}\times \left(N_{k}-3\right)/2+3\times 3\times C_{in}\times C_{out}$	W_in×H_in×3×3×C_in×((N_k−3))/2+W_in×H_in×3×3×C_in×C_out	$\frac{9\times \left(N_{k}-3\right)}{2N_{k}^{2}\times C_{out}}+\frac{9}{N_{k}^{2}}$

**Table 2:** Detailed information of the MSTAR dataset.

Type	Serial Number	Number of Samples	
		17° Depression	15° Depression
2S1	b01	299	274
BMP-2	9563	233	195
	9566	232	196
	c21	233	196
BRDM-2	E-71	298	274
BTR-60	K10yt7532	256	195
BTR-70	c71	233	196
D7	92v13015	299	274
T-62	A51	299	273
T-72	132	232	196
	812	231	195
	s7	233	191
ZIL-131	E12	299	274
ZSU-234	d08	299	274

**Table 3:** The main structure of the MSCAE with four modality levels utilized in the experiments.

Stage	Level	Input Size	Processes	Output Size	Feature Size
Encoder	1	128×128×1	$\left(\begin{array}{c} CSeConv,5\times 5\times 1,\;8,stride\;2\\ BN+ReLU\\ PPM\;Block \end{array}\right)$	64×64×8	$\left(\begin{array}{c} 64\times 64\times 1\\ 32\times 32\times 8\\ 16\times 16\times 8\\ 1\times 1\times 8 \end{array}\right)$
	2	64×64×8	$\left(\begin{array}{c} Max\;Pooling,\;2\times 2\\ CSeConv,\;5\times 5\times 8,\;16,\;stride\;2\\ BN+ReLU\\ PPM\;Block \end{array}\right)$	16×16×16	$\left(\begin{array}{c} 16\times 16\times 1\\ 8\times 8\times 16\\ 4\times 4\times 16\\ 1\times 1\times 16 \end{array}\right)$
	3	16×16×16	$\left(\begin{array}{c} Max\;Pooling,\;2\times 2\\ CSeConv,\;5\times 5\times 16,\;32,\;stride\;2\\ BN+ReLU\\ PPM\;Block \end{array}\right)$	4×4×32	$\left(\begin{array}{c} 4\times 4\times 1\\ 2\times 2\times 32\\ 1\times 1\times 32 \end{array}\right)$
	4	4×4×32	$\left(\begin{array}{c} Max\;Pooling,\;2\times 2\\ Vectorization \end{array}\right)$	2×2×32	128×1×1
Decoder	4	128×1×1	Reshape	2×2×32	-
	3	$\left(\begin{array}{c} 2\times 2\times 32\\ 176\times 1\times 1 \end{array}\right)$	$\left(\begin{array}{c} FAM\;Block\\ CSeDeConv,\;5\times 5\times 64,\;16,\;Stride\;2\\ BN+ReLU \end{array}\right)$	8×8×16	-
	2	$\left(\begin{array}{c} 8\times 8\times 16\\ 1152\times 1\times 1 \end{array}\right)$	$\left(\begin{array}{c} FAM\;Block\\ CSeDeConv,\;5\times 5\times 32,\;8,\;Stride\;2\\ BN+ReLU \end{array}\right)$	32×32×8	-
	1	$\left(\begin{array}{c} 32\times 32\times 8\\ 14344\times 1\times 1 \end{array}\right)$	$\left(\begin{array}{c} FAM\;Block\\ CSeDeConv,\;5\times 5\times 16,\;4,\;Stride\;2\\ CSeDeConv,\;3\times 3\times 4,\;1,\;Stride\;1\\ Sigmoid \end{array}\right)$	128×128×1	-

**Table 4:** Classification results on the three-target dataset.

Scheme	BMP-2	BTR-70	T-72	Without Variants	Variants Only	Average $P_{cc}$
L 1	89.05%	99.39%	99.79%	96.15%	94.34%	95.12%
L 2	87.01%	98.88%	96.02%	95.13%	92.28%	92.56%
L 3	82.35%	98.06%	96.01%	93.08%	90.37%	90.44%
L 4	77.92%	93.88%	96.80%	90.23%	86.78%	88.26%
L 1+2	95.33%	99.39%	99.14%	98.47%	97.35%	97.54%
L 1+3	94.52%	98.98%	99.38%	98.30%	96.95%	97.24%
L 1+4	93.94%	99.39%	99.62%	98.26%	96.92%	97.14%
L 2+3	96.13%	97.76%	96.70%	97.55%	96.27%	96.61%
L 2+4	93.52%	98.98%	99.48%	97.75%	96.74%	96.85%
L 3+4	92.22%	98.67%	99.48%	97.38%	96.05%	96.25%
L 1+2+3	96.70%	99.39%	99.90%	99.25%	98.15%	98.45%
L 1+2+4	95.57%	99.39%	99.79%	98.91%	97.62%	97.92%
L 1+3+4	95.30%	99.39%	99.90%	98.26%	97.53%	97.85%
L 2+3+4	95.06%	99.18%	99.90%	98.50%	97.54%	97.71%
All	99.05%	99.69%	99.04%	99.73%	98.69%	99.14%

**Table 5:** Validation of the components in the proposed MSCAE model.

Model	Description	BMP-2	BTR-70	T-72	$P_{cc}$
No. 1	No PPM and FAM	93.41%	98.81%	99.66%	96.85%
No. 2	No CSeConv and CSeDeConv	97.33%	99.66%	99.83%	98.73%
No. 3	MSE Measurement	93.98%	99.83%	99.83%	97.31%
No. 4	No ILSF	95.17%	99.15%	99.71%	97.68%
No. 5	No Speckle Suppression Restriction	96.15%	99.49%	99.60%	98.10%
Baseline	The proposed MSCAE	99.05%	99.69%	99.04%	99.14%

**Table 6:** Classification results of the ten-target MSTAR dataset.

Scheme	2S1	BMP-2	BRDM-2	BTR-60	BTR-70	D7	T-62	T-72	ZIL-131	ZSU-234	$P_{cc}$
L. 1	95.3%	89.8%	93.1%	96.9%	99.0%	98.2%	95.6%	99.8%	98.5%	99.3%	96.1%
L. 2	88.7%	86.2%	88.3%	93.3%	94.4%	93.8%	89.7%	97.8%	98.2%	98.9%	92.7%
L. 3	83.2%	91.8%	87.6%	88.2%	93.4%	94.2%	85.7%	95.4%	94.9%	96.7%	91.6%
L. 4	75.5%	85.2%	81.8%	81.5%	91.8%	94.5%	83.2%	92.8%	79.9%	97.8%	86.9%
L. 1+2	94.9%	95.7%	93.8%	97.9%	98.5%	99.3%	96.3%	99.8%	99.3%	100.0%	97.6%
L. 1+3	96.7%	95.9%	94.2%	99.5%	99.5%	99.6%	95.6%	99.8%	98.5%	99.6%	97.8%
L. 1+4	97.1%	93.9%	94.2%	97.9%	99.0%	100.0%	97.4%	99.8%	98.5%	99.6%	97.5%
L. 2+3	92.7%	91.8%	97.4%	97.4%	99.5%	98.2%	93.4%	98.8%	98.2%	99.3%	95.7%
L. 2+4	90.9%	92.3%	90.9%	95.9%	98.5%	97.8%	94.1%	97.8%	97.8%	98.9%	95.3%
L. 3+4	86.5%	93.9%	92.3%	92.3%	96.9%	97.8%	89.7%	96.9%	94.5%	97.1%	94.1%
L. 1+2+3	96.7%	97.6%	96.4%	99.0%	99.5%	99.6%	97.1%	99.8%	98.9%	100.0%	98.5%
L. 1+2+4	96.7%	97.4%	95.3%	97.9%	99.5%	99.6%	97.4%	99.7%	99.3%	100.0%	98.3%
L. 1+3+4	96.7%	98.0%	96.0%	98.5%	99.5%	99.6%	96.7%	99.8%	98.5%	99.6%	98.4%
L. 2+3+4	94.2%	93.4%	98.5%	99.5%	99.5%	98.5%	93.0%	98.3%	97.8%	99.3%	96.2%
ALL	99.3%	98.3%	98.5%	99.0%	99.5%	99.6%	98.5%	99.8%	99.3%	100.0%	98.9%
